# Incidence and Outcomes of Bloodstream Infection After Arterial Aneurysm Repair: Findings From a Population-Based Study

**DOI:** 10.1093/ofid/ofad521

**Published:** 2023-10-21

**Authors:** Hussam Tabaja, Larry M Baddour, Supavit Chesdachai, Randall R DeMartino, Brian D Lahr, Daniel C DeSimone

**Affiliations:** Division of Public Health, Infectious Diseases and Occupational Medicine, Department of Medicine, Mayo Clinic, Rochester, Minnesota, USA; Division of Public Health, Infectious Diseases and Occupational Medicine, Department of Medicine, Mayo Clinic, Rochester, Minnesota, USA; Department of Cardiovascular Diseases, Mayo Clinic, Rochester, Minnesota, USA; Division of Public Health, Infectious Diseases and Occupational Medicine, Department of Medicine, Mayo Clinic, Rochester, Minnesota, USA; Department of Vascular Surgery, Mayo Clinic, Rochester, Minnesota, USA; Division of Clinical Trials and Biostatistics, Department of Quantitative Health Sciences, Mayo Clinic, Rochester, Minnesota, USA; Division of Public Health, Infectious Diseases and Occupational Medicine, Department of Medicine, Mayo Clinic, Rochester, Minnesota, USA; Department of Cardiovascular Diseases, Mayo Clinic, Rochester, Minnesota, USA

**Keywords:** aneurysm, bloodstream infection, endovascular, graft, incidence

## Abstract

**Background:**

Limited research has focused on bloodstream infection (BSI) in patients with arterial grafts. This study aims to describe the incidence and outcomes of BSI after arterial aneurysm repair in a population-based cohort.

**Methods:**

The expanded Rochester Epidemiology Project (e-REP) was used to analyze aneurysm repairs in adults (aged ≥18 years) residing in 8 counties in southern Minnesota from January 2010 to December 2020. Electronic records were reviewed for the first episode of BSI following aneurysm repair. BSI patients were assessed for vascular graft infection (VGI) and followed for all-cause mortality.

**Results:**

During the study, 643 patients had 706 aneurysm repairs: 416 endovascular repairs (EVARs) and 290 open surgical repairs (OSRs). Forty-two patients developed BSI during follow-up. The 5-year cumulative incidence of BSI was 4.7% (95% confidence interval [CI], 3.0%–6.4%), with rates of 4.0% (95% CI, 1.8%–6.2%) in the EVAR group and 5.8% (95% CI, 2.9%–8.6%) in the OSR group (*P* = .052). Thirty-nine (92.9%) BSI cases were monomicrobial, 33 of which were evaluated for VGI. VGI was diagnosed in 30.3% (10/33), accounting for 50.0% (8/16) of gram-positive BSI cases compared to 11.8% (2/17) of gram-negative BSI cases (*P* = .017). The 1-, 3-, and 5-year cumulative post-BSI all-cause mortality rates were 22.2% (95% CI, 8.3%–34.0%), 55.8% (95% CI, 32.1%–71.2%), and 76.8% (95% CI, 44.3%–90.3%), respectively.

**Conclusions:**

The incidence of BSI following aneurysm repair was overall low. VGI was more common with gram-positive compared to gram-negative BSI. All-cause mortality following BSI was high, which may be attributed to advanced age and significant comorbidities in our cohort.

## Teaser

Bloodstream infection (BSI) after arterial aneurysm repair is uncommon. Vascular graft infection is more likely following gram-positive compared to gram-negative BSI. High all-cause mortality results from advanced age and multiple comorbidities in these patients.

Vascular reconstructive surgery has revolutionized the management of arterial aneurysms, resulting in improved prognosis since its introduction in the 1950s [1]. Nonetheless, patients with a vascular graft are prone to infectious complications due, in part, to older age, underlying multiple comorbidities, recurrent healthcare exposures, and interventions. Understanding the natural history of these complications is paramount to enhancing postoperative care in patients with vascular grafts.

We recently utilized the expanded Rochester Epidemiology Project (e-REP) to conduct a population-based investigation including all patients undergoing aneurysm repairs residing in 8 counties in southern Minnesota from 2010 through 2020. In a previous publication, we described the incidence of vascular graft infection (VGI) in the original population of 708 repairs [2]. Overall, the incidence of VGI was low, with a 5-year cumulative incidence of 1.6% (95% confidence interval [CI], .6%–2.7%) and with no difference between endovascular repair (EVAR) and open surgical repair (OSR) (1.4% vs 2.0%; *P* = .843). In addition, we discussed trends in the type of aneurysm repair procedures performed over the 10-year period and compared the baseline characteristics of patients who underwent EVAR with those who underwent OSR. Survival following aneurysm repair, as well as survival following VGI, was also evaluated.

Herein, we aim to describe the natural history of bloodstream infection (BSI) following aneurysm repair. In this setting, determining the risk of VGI is the biggest challenge associated with BSI. The incidence of BSI and its consequences in this group of patients has received scant attention. As such, the objective of the current study is to use contemporary population-based data to describe the incidence, epidemiology, and outcome of the initial episode of BSI following arterial aneurysm repair.

## METHODS

### Study Participants and Data Collection

The e-REP was queried for all adults (aged ≥18 years) residing in 8 counties (Olmsted, Mower, Goodhue, Freeborn, Steele, Wabasha, Dodge, and Waseca) in southern Minnesota who underwent arterial aneurysm repair between 1 January 2010 and 31 December 2020. The e-REP medical chart linkage system was described with citation in our prior publication [2–4]. We used the *International Classification of Diseases, Ninth* and *Tenth Revisions*, and the *Current Procedural Terminology* diagnostic and procedure codes for arterial aneurysm and arterial aneurysm repair to retrieve our patient list (billing codes available in prior publication [2]). Inclusion was limited to these 8 counties because the e-REP provided coverage for at least 90% of their residents throughout most of the study period [5]. We excluded patients who underwent arterial repair for conditions other than aneurysms and patients who declined the Minnesota research authorization.

The Mayo Clinic is the only hospital system that performs aneurysm repairs in the studied region. Therefore, all aneurysm repairs of the studied population were performed at the Mayo Clinic in Rochester and the Mayo Clinic Health System. Some patients underwent several aneurysm repairs during the study period; hence, we collected and analyzed data for all repair procedures. From the time of each index procedure (first repair at the aneurysm site), patients were followed up for development of first BSI to calculate the incidence, as described in the “Follow-up” section of the Supplementary material. Patients with BSI were then evaluated for 2 outcomes. The first outcome was a diagnosis of VGI, which was assessed in patients with at least 14 days of post-BSI follow-up to exclude early dropouts. The second outcome was all-cause mortality.

Clinical variables including patient demographics, type of aneurysm repair procedure, diagnosis of BSI, diagnosis of VGI, microbiology, treatment, and outcomes were manually abstracted through medical chart review. The study was approved by the Mayo Clinic and Olmsted Medical Center institutional review boards (IRB-21002700). Data collection was completed on 30 July 2022.

### Definitions

#### Arterial Aneurysm Repair

Arterial aneurysm repair was defined as the use of a graft during OSR or a stent-graft during EVAR to fix arterial aneurysmal dilatation. Hereafter, the term “graft” refers to both open surgical graft and endovascular stent-graft, unless otherwise specified. We categorized aneurysms into intracavitary and extracavitary. The former group included the thoracic aorta, thoracoabdominal aorta, abdominal aorta, subclavian artery, and iliac artery. The latter included the femoral artery and popliteal artery. The common, external, and internal iliac arteries were grouped under the iliac artery category. The common, superficial, and profunda femoral arteries were grouped under the femoral artery category. Aneurysms of the thoracic aorta were further distinguished into ascending thoracic aneurysms—including root and arch—and descending thoracic aneurysms. All ascending thoracic aneurysms were repaired by cardiovascular surgeons through OSR. All remaining aneurysms, including those of descending thoracic aorta, were repaired by vascular surgeons with either OSR or EVAR or interventional radiologists with EVAR.

#### Bloodstream Infection

BSI was defined as the detection of bacterial or fungal growth in at least 1 bottle within a blood culture set, which was not attributed to contamination. Contaminated blood cultures were defined as any of (1) discordant growth of common contaminants in only 1 bottle out of multiple without prior antimicrobial exposure [6], such as coagulase-negative staphylococci, *Corynebacterium* spp, *Micrococcus* spp, *Bacillus* spp other than *anthracis*, viridans group streptococci, *Cutibacterium* spp, and *Clostridium perfringens* [7]; or (2) positive blood cultures from an indwelling arterial or venous catheter in the absence of positive peripheral blood cultures [6].

Time to BSI was measured from date of index aneurysm repair to date of first positive blood culture bottle. BSI was considered “early” if it occurred ≤30 days from index aneurysm repair. Furthermore, BSI was classified as community-onset or nosocomial [8, 9]. Community-onset was defined as positive blood cultures obtained at the time of admission or within 48 hours of admission while nosocomial was defined as positive blood cultures obtained after 48 hours of admission. Community-onset was further classified into community-acquired or healthcare-associated based on previously defined criteria [8]. The duration of BSI was defined as the number of days from first positive blood culture bottle to the last positive follow-up blood culture bottle. For patients without follow-up blood cultures, the duration of BSI was recorded as 1 day. Persistent BSI was defined as positive follow-up blood cultures ≥24 hours after starting effective antibiotics based on in vitro susceptibilities [10], and prolonged BSI was defined as positive blood cultures for ≥72 hours [9].

#### Vascular Graft Infection

VGI was defined based on criteria published by the Management of Aortic Graft Infection Collaboration (MAGIC), which classifies VGI diagnosis into “confirmed” and “suspected” [11]. To establish the temporal relationship between BSI and VGI, we followed a similar approach to that used by Fang et al to study the association between BSI and endocarditis [12]. Specifically, any VGI diagnosed within 14 days of BSI would classify as VGI at the outset. In this situation, it is not possible to determine whether VGI was the cause or the result of BSI. Conversely, any VGI diagnosed ≥14 days after BSI would classify as a new-onset VGI, likely caused by BSI resulting in graft seeding.

### Statistical Analysis

Analyses were performed on repair procedures as the observational units, with some patients contributing multiple observations. Baseline characteristics are presented as median (interquartile range [IQR]) for continuous variables and frequency (percentage) for categorical variables. For post-repair BSI and post-BSI mortality outcomes, time-to-event analyses were conducted to account for variable lengths of follow-up. The analyses for time to BSI considered death as a competing risk event using cumulative incidence function estimates and proportional subdistribution hazards regression. For the latter analysis, we fit an unadjusted model to compare the cumulative risk of BSI between the OSR and EVAR groups, accounting for correlated responses from the same patient using the Huber-White cluster sandwich estimator [13]. Mortality was analyzed using the Kaplan-Meier estimator, while follow-up time was summarized using the reverse Kaplan-Meier method (ie, time to censoring). Analyses were conducted using R version 4.0.3 software (R Foundation, Vienna, Austria).

## RESULTS

**Figure 1.**
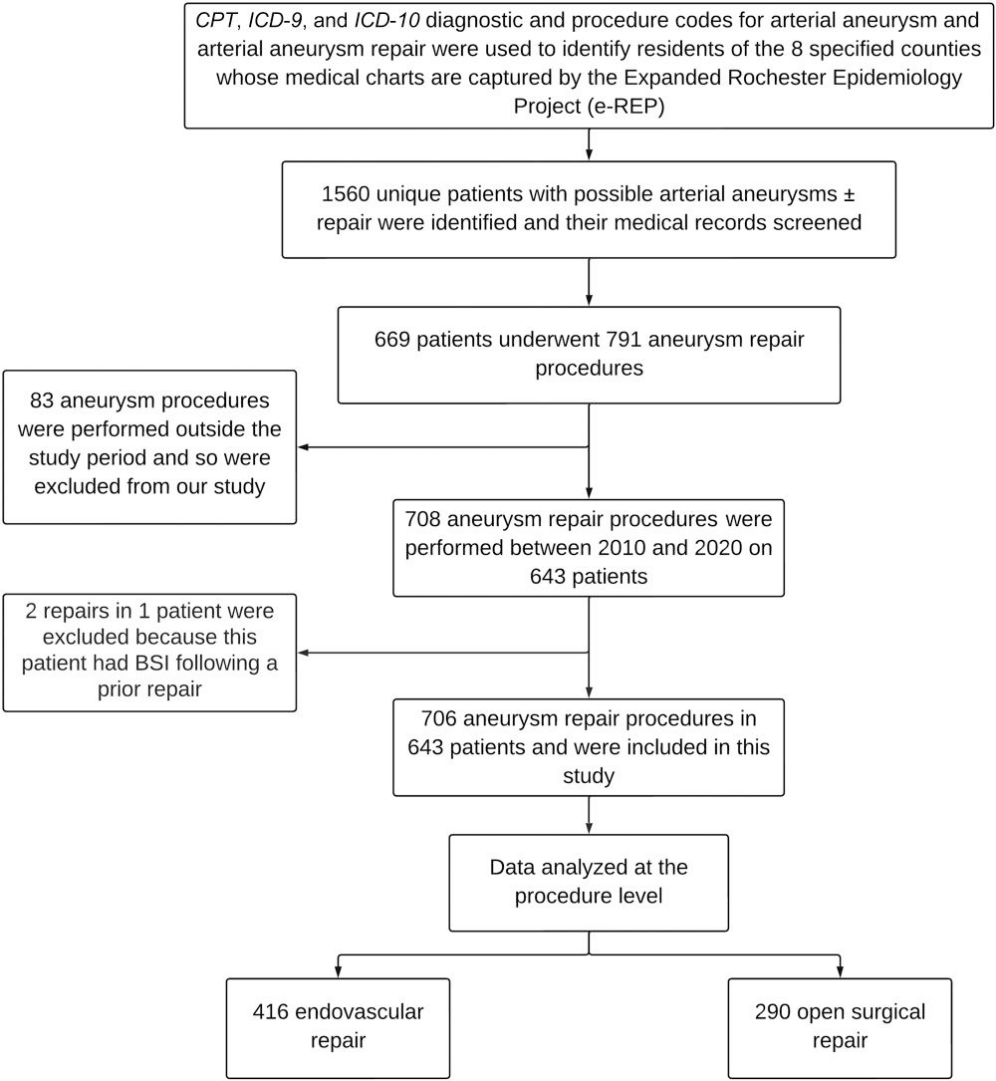
Inclusion flowchart. Abbreviations: BSI, bloodstream infection; CPT, *Current Procedural Terminology*; *ICD-9*, *International Classification of Diseases, Ninth Revision*; *ICD-10*, *International Classification of Diseases, Tenth Revision*.

As described above, our original cohort from the prior study on incidence of VGI included 708 repair procedures [2]. However, with BSI as the primary endpoint in the current investigation, we excluded 2 repairs that were performed after the patient's prior repair had resulted in BSI, at which time their procedural follow-up was terminated. Therefore, for this current study, 643 patients who underwent a total of 706 aneurysm repair procedures were included: 290 (41.1%) OSR and 416 (58.9%) EVAR procedures (see Figure 1 for inclusion flowchart). The median patient age at the time of aneurysm repair was 74.1 (IQR, 66.3–80.5) years. Overall, 561 (79.5%) procedures were performed in male patients and 682 (96.9%) in White patients. The Charlson Comorbidity Index (CCI) score was elevated with a median of 6 (IQR, 4–8). Table 1 summarizes baseline comorbidities of the study population. Baseline differences between OSR and EVAR procedures have been previously reported [2], demonstrating that patients undergoing OSR were younger, generally with fewer comorbidities, but more likely to have heart valve disease.

**Table 1.** Baseline Comorbidities of Study Population at Index Aneurysm Repair

Characteristic	(N = 706)
Age at time of repair, y	74.1 (66.3–80.5)
Sex, male	561 (79.5)
Race, White	682 (96.9)
BMI, kg/m^2^	28.9 (25.7–33.0)
Coronary artery disease	506 (71.7)
Coronary intervention	210 (29.7)
Congestive heart failure	208 (29.5)
Heart valve disease	435 (61.6)
Cerebrovascular accident or transient ischemic attack	235 (33.3)
Connective tissue disease	61 (8.6)
Liver disease	163 (23.1)
Diabetes mellitus	201 (28.5)
Chronic kidney disease, moderate to severe	50 (7.1)
Cancer	73 (10.3)
Transplant	9 (1.3)
Cardiovascular devices	
No. of devices	
0	519 (73.5)
1	155 (22.0)
≥2	32 (4.5)
Valve prosthesis	63 (8.9)
CIED	40 (5.7)
Prior aneurysm repair	106 (15.0)

Values represent frequency (%) for categorical variables and median (interquartile range) for continuous variables.

Abbreviations: BMI, body mass index; CIED, cardiovascular implantable electronic device.

There were 666 (94.3%) intracavitary aneurysm repair procedures, including the ascending thoracic aorta in 132 cases, subclavian artery in 2, descending thoracic aorta in 25, thoracoabdominal aorta in 42, abdominal aorta in 438, and iliac artery in 27. The remaining 40 (5.7%) procedures were extracavitary aneurysm repairs, which involved the femoral artery in 8 cases and the popliteal artery in 32 cases. Eighty-five (12.0%) repair cases were considered emergent, due to acute dissection with rupture in 50 cases, impending rupture in 31, and critical limb ischemia in 4.

### Incidence of First BSI Following Arterial Aneurysm Repair

**Figure 2.**
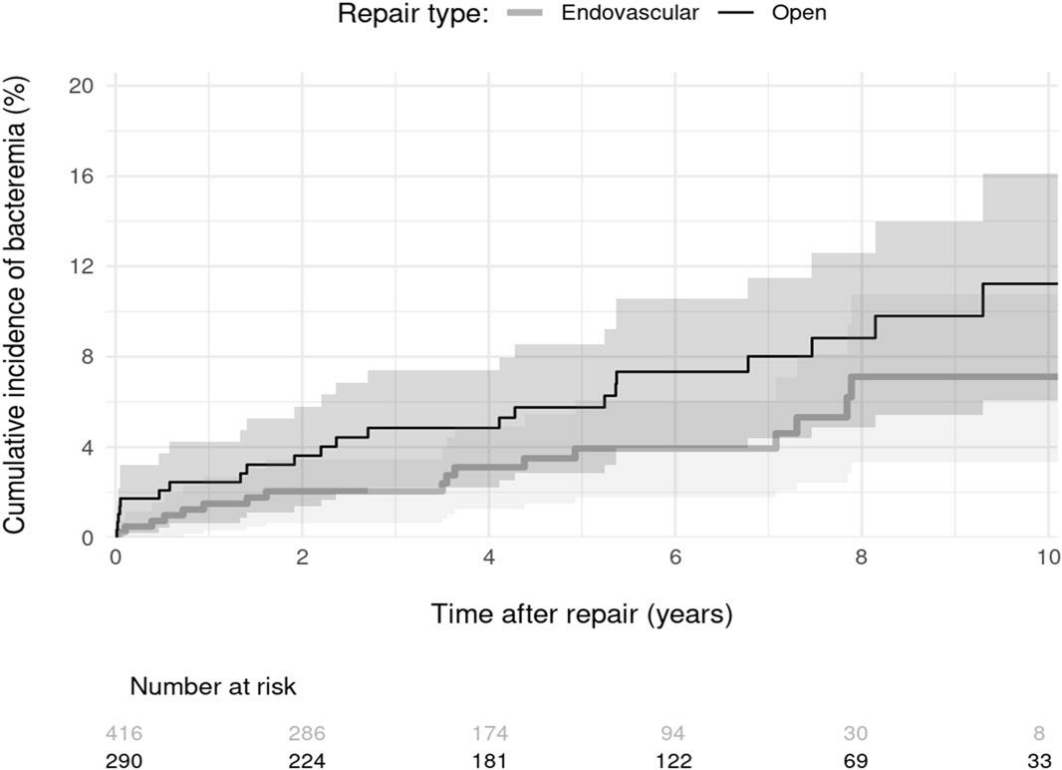
Cumulative incidence of bloodstream infection over time from index arterial aneurysm repair.

During a median follow-up of 4.2 (IQR, 1.9–6.8) years from index repair, 42 patients developed BSI, corresponding to a BSI rate of 13.6 events per 1000 procedure-years (Table 2). All BSI events included bacterial pathogens with no cases of fungemia detected during follow-up. The median time from the index arterial aneurysm repair to BSI was 3.1 (IQR, 0.6–6.4) years. Only 6 (14.3%) patients had early BSI (ie, ≤30 days following aneurysm repair), of which 5 belonged to the OSR group. Furthermore, BSI occurred during the same admission for index aneurysm repair in 3 (7.1%) patients, all belonging to the OSR group. Overall, the 30-day cumulative incidence of BSI was 0.9% (95% CI, .2%–1.5%), and the cumulative event rate at 5-year follow-up was 4.7% (95% CI, 3.0%–6.4%). The cumulative incidence of BSI was marginally higher (subdistribution hazard ratio, 1.86 [95% CI, 1.00–3.49]; *P* = .052) in the OSR group than in the EVAR group (5-year rate: 5.8% vs 4.0%), although the difference between the 2 curves appeared very early on (eg, 30-day rate: 1.7% vs .2%) and plateaued thereafter (Figure 2).

**Table 2.** Cumulative Incidence of Bloodstream Infection

Incidence	Overall (N = 706)	Endovascular (n = 416)	Open (n = 290)
Incident BSI cases, No.	42	17	25
Procedure-years	3093.7	1568.0	1525.6
Incidence rate/1000 procedure-years (95% CI)	13.6 (9.8–18.4)	10.8 (6.3–17.4)	16.4 (10.6–24.2)
Cumulative incidence, % (95% CI)			
30 d	0.9% (.2%–1.5%)	0.2% (.0%–.7%)	1.7% (.2%–3.2%)
5 y	4.7% (3.0%–6.4%)	4.0% (1.8%–6.2%)	5.8% (2.9%–8.6%)
sHR (95% CI)	…	1.0 (ref)	1.86 (1.00–3.49)

sHR was estimated by proportional subdistribution hazards regression to take into account the competing risk of death, used in conjunction with the Huber-White cluster sandwich estimator to correct model estimates for correlated responses from the same patient.

Abbreviations: BSI, bloodstream infection; CI, confidence interval; sHR, subdistribution hazard ratio.

The median age at the time of procedure for the BSI subgroup was 74.2 (IQR, 67.0–78.9) years. The median CCI score was 6 (IQR, 4.3–8.0). Supplementary Table 1 summarizes the baseline characteristics of the BSI group, and Supplementary Table 2 provides a more detailed account of each patient. There were 25 (59.5%) OSR and 17 (40.5%) EVAR procedures. This included 41 (97.6%) intracavitary aneurysm repairs, including ascending thoracic aorta in 13 patients, descending thoracic aorta in 3, thoracoabdominal aorta in 1, and abdominal aorta in 24. There was only 1 (2.4%) patient with extracavitary repair procedure including the femoral artery. Furthermore, in 5 (11.9%) patients, the repair procedure was considered emergent.

BSI was considered community-onset in 36 (85.7%) patients, including community-acquired in 16 and healthcare-associated in 20. The remaining 6 (14.3%) patients had nosocomial BSI. The median number of positive blood culture sets and positive blood culture bottles was 2.0 (IQR, 1.0–4.0) and 4.0 (IQR, 2.0–9.5), respectively. In 37 (88.1%) patients, follow-up blood cultures were obtained to document clearance. The median duration of BSI was 1.0 (IQR, 1.0–2.0) day. BSI was persistent in 13 (31.0%) and prolonged in 10 (23.8%) patients.

**Figure 3.**
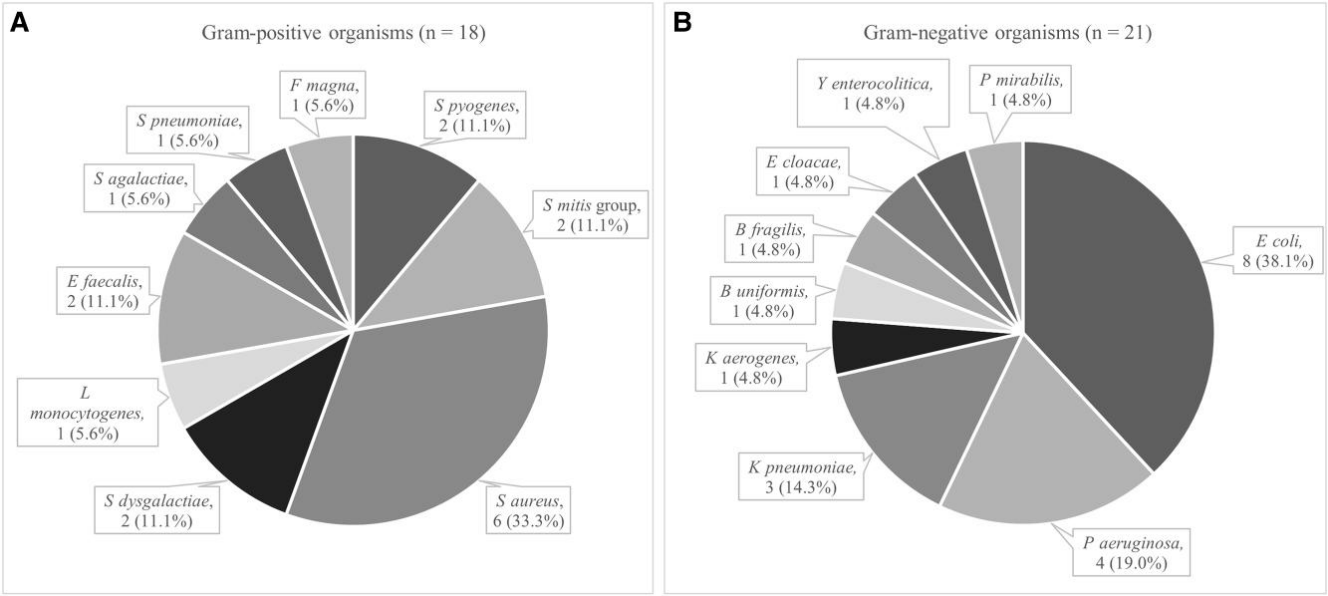
Microbial distribution for monomicrobial bloodstream infection (BSI) cases (n = 39). *A*, Microbial distribution for gram-positive monomicrobial BSI. *B*, Microbial distribution for gram-negative monomicrobial BSI.

**Figure 4.**
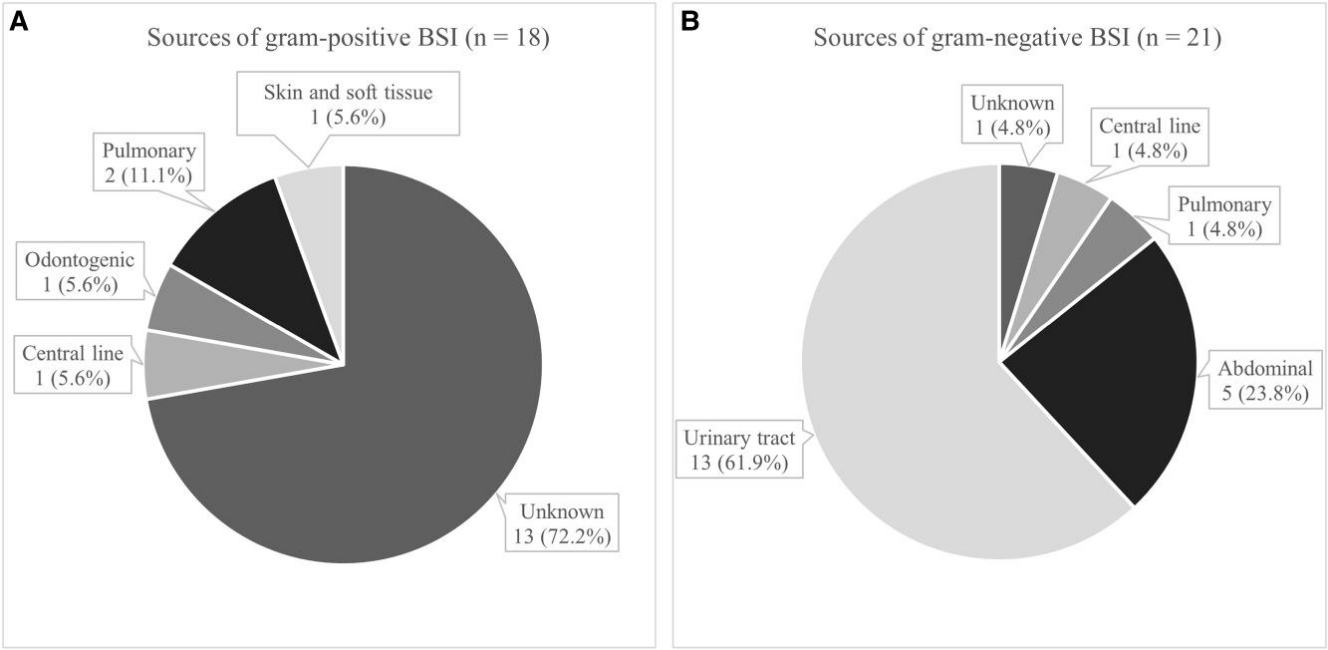
Sources of monomicrobial bloodstream infection (BSI) (n = 39). *A*, Sources for monomicrobial gram-positive BSI. *B*, Sources for monomicrobial gram-negative BSI. “Pulmonary” includes infection of the lungs and pleura. “Abdominal” includes infections of the gastrointestinal tract, biliary tract, and peritoneum.

Thirty-nine (92.9%) patients had monomicrobial BSI while 3 (7.1%) had polymicrobial BSI. Of the 39 patients with monomicrobial BSI, 18 (46.2%) had gram-positive organisms and 21 (53.8%) had gram-negative organisms. Figure 3 shows the microbial distribution for the monomicrobial BSI cases. *Streptococcus* spp were the most common gram-positive organisms followed by *Staphylococcus aureus*. In the gram-negative group, *Escherichia coli* was the most frequent organism. Table 3 compares gram-positive and gram-negative monomicrobial BSI cases, and Figure 4 outlines their primary sources. Compared to gram-negative BSI, gram-positive BSI was more likely to have an unidentified primary source. Furthermore, gram-positive BSI cases were more persistent and had a higher number of positive blood culture sets and bottles.

**Table 3.** Characteristics of Monomicrobial Bloodstream Infection According to Gram Type (n = 39)

Characteristic	Gram-Positive (n = 18)	Gram-Negative (n = 21)
Community-onset	17 (94.4)	17 (81.0)
Community-acquired	10 (55.6)	5 (23.8)
Healthcare-associated	7 (38.9)	12 (57.1)
Nosocomial	1 (5.6)	4 (19.0)
Duration of BSI, d	2.0 (1.0–4.8)	1.0 (1.0–1.0)
Prolonged BSI	8 (44.4)	2 (9.5)
No. of positive blood culture sets	4.0 (1.3–6.5)	2.0 (1.0–2.0)
No. of positive blood culture bottles	9.0 (2.8–12.2)	3.0 (2.0–5.0)

Values represent frequency (%) for categorical variables and median (interquartile range) for continuous variables.

Abbreviation: BSI, bloodstream infection.

### Rates of VGI in the Setting of First BSI Event

Thirty-three of the 39 (84.6%) patients with monomicrobial BSI completed ≥14 days of follow-up after their first BSI event. Advanced imaging was conducted at the onset of BSI in 16 (48.5%) patients to evaluate graft infection. This included computed tomography with angiogram (CT-A) in 9 patients and nuclear scans in 13 patients, with some individuals undergoing both types of imaging. Nuclear scans included (18)F-fluorodeoxyglucose positron emission tomography with CT in 8 and indium-labelled white blood cell scan (I^111^) in 6, with 1 patient undergoing both. Of these 16 patients, 12 (75.0%) had gram-positive BSI, 10 (62.5%) had persistent BSI, and 10 (62.5%) had an undetermined primary source. Furthermore, all 15 patients had community-onset BSI, including 8 community-acquired and 7 healthcare-associated. VGI occurred in 10 (30.3%) of the 33 patients. All VGI cases were diagnosed within 14 days of incident BSI (ie, VGI at the outset of BSI). The diagnosis was considered confirmed in 6 and suspected in 4. Supplementary Table 3 specifies the criteria met by each patient.

In the 10 VGI patients, the median time from index repair to first BSI event (median, 1.5 [IQR, 1.0–4.6] years) was 2 years shorter than that (median, 3.5 [IQR, 0.4–5.3] years) of the 23 patients without VGI, although the overall difference was not statistically significant (*P* = .938). All 10 patients with VGI had community-onset BSI, and for 6 of 10 the primary source was undetermined. In this subgroup, BSI was persistent in 7 (70.0%) patients and prolonged in 4 (40.0%); the median BSI duration was 2.0 (IQR, 1.2–4.5) days. The median number of positive blood culture sets and bottles was 4.0 (IQR, 2.2–4.8) and 9.0 (IQR, 5.0–10.0), respectively. The median BSI duration for the 23 patients without VGI was 1.0 (IQR, 1.0–1.5) days, which was significantly shorter than that of the VGI group (*P* = .025). Eight VGIs events occurred following gram-positive BSI and 2 following gram-negative BSI. Monomicrobial gram-positive BSI was associated with a higher rate of VGI compared to that of monomicrobial gram-negative BSI (50.0% vs 11.8%, *P* = .017).

Only 3 (30.0%) patients with VGI had surgical explantation of an infected graft, 2 of whom had positive cultures matching a blood culture isolate and 1 of whom had negative graft cultures possibly due to recent antibiotic exposure. A fourth patient had image-guided aspiration of perigraft fluid with the same pathogen as recovered from blood cultures. The remaining 6 patients were managed conservatively and had no graft or perigraft cultures done (Supplementary Table 4).

Six (15.4%) patients with monomicrobial BSI did not complete a 14-day post-BSI follow-up and so were not evaluated for VGI; 5 patients died as described in the survival section and 1 patient was lost to follow-up. In these 6 patients, BSI was due to gram-positive organisms in 3 and gram-negative organisms in 4.

### Survival Following BSI

A total of 22 patients died during a median follow-up time of 2.7 (IQR, 1.3–6.0) years from incident BSI, of which 6 (27.3%) deaths were related to BSI. The 30-day mortality rate was 12.1%, and all 5 of these deaths were attributable to BSI. Furthermore, the cumulative all-cause mortality rates at 1, 3, and 5 years were 22.2% (95% CI, 8.3%–34.0%), 55.8% (95% CI, 32.1%–71.2%), and 76.8% (95% CI, 44.3%–90.3%), respectively.

## DISCUSSION

Our study provides insight into the incidence and epidemiology of BSI following arterial aneurysm repair. The incidence of first BSI event following repair in our population was overall low. Although there was a signal for a lower BSI incidence following EVAR compared to OSR, this was not statistically significant. The BSI events were typically delayed, occurring at a median of 3 years from index procedure. Early BSI was seen in a small group of patients, most of whom belonged to the OSR group. A majority of patients had community-onset and monomicrobial BSI with similar rates of gram-positive and gram-negative organisms. Moreover, more than half of the community-onset BSIs were healthcare-associated. The primary source for BSI was undetermined in one-third of cases, particularly more common with gram-positive as compared to that with gram-negative BSI. VGI was diagnosed in one-third of patients with monomicrobial BSI. All VGI cases occurred at the outset of BSI. Gram-positive BSI had a significantly higher rate of VGI as compared to that due to gram-negative BSI. Finally, a high all-cause mortality following BSI was demonstrated.

To our knowledge, our study is the only population-based investigation evaluating the incidence of BSI following arterial aneurysm repair. The only prior population-based study in the English literature focused on periprocedural nosocomial BSI occurring during the same admission for the index aneurysm repair [14]. In that study, Vogel et al reported a 1.6% periprocedural BSI rate following abdominal aortic aneurysm repair (AAA) in 13 902 patients in Washington State between 1987 and 2005. The BSI rate depicted in their study was much lower following EVAR as compared to that of OSR (0.7% vs 1.6%, respectively). In contrast, our study was not restricted to periprocedural BSI nor to AAA but included first incident BSI occurring at any time following repair of an arterial aneurysm. Only 3 of our patients experienced BSI during the same admission for index aneurysm repair, which indicates a lower rate compared to that reported by Vogel et al [14]. Furthermore, the 30-day cumulative BSI incidence in our cohort was 0.9%. The lower periprocedural BSI detected in our investigation may indicate improved preventive measures and periprocedural care. Furthermore, the higher rate of EVAR procedures in our cohort compared to the other study (58.9% vs 9.2%) may have impacted the early BSI rates in our study. While we did not prove a statistically significant difference in all-time BSI following EVAR and OSR, it remains plausible that EVAR may be associated with lower rates of early BSI as seen in their study. In fact, we did detect a marginally higher rate of early BSI following OSR compared to EVAR. We found that 5 of 6 patients who experienced early BSI in our study belonged to the OSR group.

The incidence of BSI in the general population was previously examined in several studies [15–17]. The overall incidence rate for BSI was 307 cases per 100 000 person-years in a study from Sweden [16]. In a study from southern Minnesota, the incidence of *S aureus* BSI in the general population reached 33.9 cases per 100 000 person-years between 2006 and 2020 [17]. As expected, the rate of BSI in the general population is lower than that in our multimorbid patient population. On the other hand, our BSI incidence is close to that reported following other cardiovascular procedures [18, 19]. The crude incidence of BSI following cardiac valve repair and transcatheter aortic valve replacement (TAVR) was previously studied in southern Minnesota using e-REP data between 2010 and 2018. The BSI rate was 1671 cases per 100 000 person-years following cardiac valve repair and 1300 cases per 100 000 person-years following TAVR [18, 19]. VGI occurred in 30% of our monomicrobial BSI cases. The rate of infective endocarditis diagnosed at the offset of BSI following valve repair was 14.3%, but the majority of BSI events were due to gram-negatives in that study [18]. On the other hand, most BSI events were due to gram-positives in the TAVR study and 40% of cases developed infective endocarditis [19].

VGI was diagnosed in 10 of 33 patients with monomicrobial BSI who completed at least 14 days post-BSI follow-up. Factors that prompted further evaluation for VGI in patients with BSI with nuclear scans or dedicated CT-A included persistent gram-positive BSI, particularly if the BSI was community-onset and the primary source could not be determined. We observed a higher rate of VGI with gram-positive BSI than with gram-negative BSI. This is expected given the higher severity of gram-positive compared to gram-negative BSI in our cohort, and the higher predilection of gram-positive organisms like *Staphylococcus* spp, *Streptococcus* spp, and *Enterococcus* spp to cause infection of medical devices. Our finding is consistent with the current literature regarding the increased risk of infection of other types of cardiovascular devices by gram-positive compared to gram-negative organisms [20–23]. Since all VGI cases occurred at the outset of BSI, we are unable to determine whether VGI was the cause or the result of BSI.

Vascular reconstructive surgery has undoubtedly improved the prognosis for patients with arterial aneurysms [24–28]. However, it is important to note that older age and presence of multiple comorbid conditions, as seen in our cohort, can increase the risk of long-term morbidity and all-cause mortality, especially when facing cardiovascular or infectious complications. In the study by Vogel et al [14], the in-hospital and 1-year all-cause mortality following VGI were 18.0% and 28.0%, respectively. Using our more contemporary data, we previously reported a post-VGI all-cause mortality of 21.2% at 1 year and 76.4% at 5 years [2]. Finally, in the current study we described the all-cause mortality specifically following BSI. The high all-cause mortality seen in our BSI subgroup is not unexpected since most patients were elderly and had multiple comorbidities.

The most important limitations in our study are its retrospective design and its low number of outcome events. The limited occurrence of both BSI and VGI events constrains the precision of our estimates and precludes conducting a multivariable risk factor analysis in risk of VGI. Despite limitations, our study's population-based design helps to mitigate referral bias and provides a depiction of the natural history of BSI following aneurysm repair. As the epidemiology of vascular reconstructive surgery continues to evolve, the incidence of BSI and VGI following aneurysm repair is expected to change.

## CONCLUSIONS

The incidence of BSI following aneurysm repair in our study population was low. While the incidence seemed somewhat higher following OSR as compared to that for EVAR, the difference was not statistically significant. At the time of BSI, VGI was more commonly observed in gram-positive than gram-negative cases, especially when the BSI was persistent, and the primary source was unknown. The long-term all-cause mortality was high, which may be attributed to the advanced age and multiple comorbid conditions that profiled our cohort.

## Supplementary Material

ofad521_Supplementary_Data

## Supplementary Data

Supplementary materials are available at *Open Forum Infectious Diseases* online. Consisting of data provided by the authors to benefit the reader, the posted materials are not copyedited and are the sole responsibility of the authors, so questions or comments should be addressed to the corresponding author.

## Notes

***Author contributions.*** H. T.: conceptualization, methodology, validation, investigation, data curation, writing–original draft, writing–review and editing, and visualization. L. M. B.: conceptualization, methodology, supervision, resources, writing–original draft, and writing–review and editing. S. C.: investigation, data curation, writing–review and editing, and visualization. R. R. D.: conceptualization, methodology, supervision, and writing–review and editing. B. D. L: methodology, validation, investigation, formal analysis, writing–original draft, writing–review and editing, and visualization. D. C. D.: conceptualization, methodology, supervision, resources, writing–original draft, and writing–review and editing.

***Patient consent.*** The study does not include factors necessitating patient consent. The design of the work has been approved by the Mayo Clinic Institutional Review Board.

***Financial support.*** This work was supported by the Rochester Epidemiology Project (grant number R01-AG034676; principal investigators: Walter A. Rocca and Jennifer L. St Sauver).
