# Supplementary material for: Incidence and Outcomes of Bloodstream Infection After Arterial Aneurysm Repair: Findings From a Population-Based Study
Source: Open Forum Infect Dis. 2023 Oct 21;10(11):ofad521. doi: 10.1093/ofid/ofad521 (PMC10644795; doi:10.1093/ofid/ofad521)
Supplement: ofad521_Supplementary_Data [file ofad521_supplementary_data.docx]

| Supplementary Table 1. Baseline Comorbidities of BSI Group at Index Aneurysm Repair. | |
| --- | --- |
|  | **(N=42)** |
| Age at time of repair, years | 74.2 (67.0-78.9) |
| Gender, male | 35 (83.3%) |
| Race, white | 42 (100.0%) |
| BMI (kg/m^2^) | 30.2 (26.7-34.2) |
| Coronary artery disease, n (%) | 37 (88.1%) |
| Coronary intervention, n (%) | 17 (40.5%) |
| Congestive heart failure, n (%) | 15 (35.7%) |
| Heart valve disease, n (%) | 33 (78.6%) |
| Cerebrovascular accident or transient ischemic attack, n (%) | 14 (33.3%) |
| Connective tissue disease, n (%) | 2 (4.8%) |
| Liver disease, n (%) | 5 (11.9%) |
| Diabetes mellitus, n (%) | 17 (40.5%) |
| Chronic kidney disease, Moderate-to-severe, n (%) | 3 (7.1%) |
| Cancer, n (%) | 8 (19.0%) |
| Transplant, n (%) | 1 (2.4%) |

Values represent frequency (percentage) for categorical variables and median (quartile 1, quartile 3) for continuous variables.

|  | | Supplementary Table 2. Summary of 42 BSI Cases following Arterial Aneurysm Repair. | | | | | | | | | | | | | | | | | | |
| --- | --- | --- | --- | --- | --- | --- | --- | --- | --- | --- | --- | --- | --- | --- | --- | --- | --- | --- | --- | --- |
| ID | Age, yrs | | Aorta/artery | Cardiac device | Repair type | BSI date | BSI onset | BSI type | Gram type | Pathogen | BSI source | Years to BSI | Number (+) sets | Number (+) bottles | BSI duration, days | CT-A | PET-CT | I^111^ | ≥14 days post-BSI | VGI |
| 1 | 71 | | Abdominal | CIED | EVAR | 2017 | CA | M | GP | *S. pyogenes* | Unknown | 7.30 | 4 | 8 | 2**^b^** | Yes^(-)^ | Yes^(+)^ | No | Yes | Yes**^e^** |
| 2 | 57 | | Descending thoracic | AV | OSR | 2016 | CA | M | GP | *S. pyogenes* | Unknown | 2.37 | 2 | 5 | 1 | Yes^(-)^ | Yes^(+)^ | Yes^(+)^ | Yes | Yes**^e^** |
| 3 | 65 | | Abdominal | - | EVAR | 2014 | CA | M | GN | *B. uniformis* | Abdominal | 0.94 | 2 | 2 | 1 | No | No | Yes^(+)^ | Yes | Yes**^f^** |
| 4 | 68 | | Abdominal | - | OSR | 2021 | HCA | M | GN | *P. aeruginosa* | Urinary | 10.32 | 1 | 2 | 1 | Yes^(+)^ | Yes^(+)^ | No | Yes | Yes**^f^** |
| 5 | 79 | | Ascending thoracic | AV**^a^** | OSR | 2015 | CA | M | GP | *S. mitis group* | Odontogenic | 1.41 | 5 | 13 | 3**^b^** | Yes^(+)^ | No | No | Yes | Yes**^f^** |
| 6 | 72 | | Abdominal | - | EVAR | 2016 | HCA | M | GP | *S. dysgalactiae* | Unknown | 1.62 | 3 | 5 | 2**^b^** | Yes^(+)^ | No | Yes^(-)^ | Yes | Yes**^f^** |
| 7 | 72 | | Abdominal | - | OSR | 2015 | HCA | M | GP | *L. monocytogenes* | Unknown | 5.36 | 4 | 10 | 2**^b^** | No | Yes^(+)^ | No | Yes | Yes**^e^** |
| 8 | 56 | | Ascending thoracic | AV^a^ | OSR | 2013 | CA | M | GP | *E. faecalis* | Unknown | 1.34 | 5 | 10 | 5**^b^** | Yes^(-)^ | No | Yes^(+)^ | Yes | Yes**^e^** |
| 9 | 71 | | Abdominal | - | EVAR | 2017 | HCA | M | GP | *MSSA* | Central line | 0.53 | 23 | 43 | 8**^b^** | No | Yes^(+)^ | No | Yes | Yes**^f^** |
| 10 | 51 | | Ascending thoracic | AV^a^ | OSR | 2012 | CA | M | GP | *E. faecalis* | Unknown | 0.58 | 4 | 10 | 14**^b^** | No | Yes^(+)^ | No | Yes | Yes**^f^** |
| 11 | 79 | | Ascending thoracic | **AV** | OSR | 2020 | HCA | M | GN | *K. pneumoniae* | Abdominal | 9.30 | 2 | 3 | 1 | Yes^(-)^ | No | No | No**^c^** | No |
| 12 | 80 | | Abdominal | - | OSR | 2015 | CA | M | GP | *MSSA* | Pulmonary | 2.70 | 1 | 2 | 1 | No | No | No | No**^d^** | No |
| 13 | 81 | | Abdominal | - | OSR | 2013 | CA | M | GN | *E. coli* | Urinary | 1.92 | 1 | 1 | 1 | No | No | No | Yes | No |
| 14 | 78 | | Descending thoracic | - | OSR | 2013 | NO | M | GN | *K. aerogenes* | Urinary | 0.02 | 1 | 2 | 1 | No | No | No | Yes | No |
| 15 | 83 | | Abdominal | - | OSR | 2010 | HCA | M | GN | *B. Fragilis* | Abdominal | 0.05 | 1 | 1 | 1 | No | No | No | Yes | No |
| 16 | 74 | | Ascending thoracic | AV,  CIED | OSR | 2018 | CA | P | Both | *C. perfringens, E. casseliflavus, H. alvei* | Abdominal | 8.15 | 2 | 4 | 1 | No | No | No | Yes | No |
| 17 | 82 | | Abdominal | - | EVAR | 2021 | CA | M | GN | *E. coli* | Urinary | 3.50 | 1 | 2 | 1 | No | No | No | Yes | No |
| 18 | 74 | | Ascending thoracic | AV,  MV | OSR | 2020 | HCA | M | GN | *E. coli* | Unknown | 10.25 | 5 | 10 | 2 | No | Yes^(-)^ | No | Yes | No |
| 19 | 82 | | Abdominal | - | EVAR | 2018 | CA | M | GP | *MSSA* | Unknown | 3.64 | 17 | 32 | 9**^b^** | No | No | No | No**^d^** | No |
| 20 | 61 | | Abdominal | - | EVAR | 2019 | CA | M | GP | *S. dysgalactiae* | Unknown | 4.93 | 1 | 2 | 1 | No | No | No | Yes | No |
| 21 | 72 | | Abdominal | - | EVAR | 2018 | NO | M | GP | *MSSA* | Unknown | 7.84 | 7 | 10 | 4**^b^** | No | No | No | Yes | No |
| 22 | 66 | | Ascending thoracic | AV,  CIED | OSR | 2019 | HCA | M | GN | *E. coli* | Urinary | 7.47 | 2 | 6 | 1 | No | No | No | Yes | No |
| 23 | 77 | | Thoraco-abdominal | - | EVAR | 2018 | HCA | M | GN | *E. coli* | Central line | 1.41 | 1 | 1 | 1 | No | No | No | Yes | No |
| 24 | 83 | | Abdominal | - | OSR | 2017 | HCA | M | GN | *Y. enterocolitica* | Abdominal | 0.47 | 2 | 4 | 1 | No | No | No | Yes | No |
| 25 | 79 | | Abdominal | AV, CIED^a^ | EVAR | 2010 | CA | M | GP | *MSSA* | Unknown | 0.39 | 2 | 6 | 1 | Yes^(-)^ | No | Yes^(-)^ | Yes | No |
| 26 | 80 | | Abdominal | - | OSR | 2020 | HCA | M | GN | *P. mirabilis* | Urinary | 10.59 | 2 | 4 | 1 | No | No | No | No**^d^** | No |
| 27 | 77 | | Abdominal | - | EVAR | 2018 | HCA | M | GN | *P. aeruginosa* | Urinary | 3.55 | 5 | 7 | 3**^b^** | No | No | No | Yes | No |
| 28 | 76 | | Ascending thoracic | AV | OSR | 2019 | NO | M | GN | *E. coli* | Urinary | 4.28 | 2 | 6 | 1 | No | No | No | Yes | No |
| 29 | 67 | | Ascending thoracic | - | OSR | 2020 | CA | M | GN | *P. aeruginosa* | Urinary | 6.78 | 6 | 12 | 3**^b^** | No | Yes^(-)^ | No | Yes | No |
| 30 | 58 | | Ascending thoracic | AV | OSR | 2019 | CA | M | GN | *K. pneumoniae* | Urinary | 4.11 | 1 | 1 | 1 | No | No | No | Yes | No |
| 31 | 70 | | Femoral | - | OSR | 2021 | HCA | M | GN | *E. cloacae* | Urinary | 5.24 | 1 | 1 | 1 | No | No | No | Yes | No |
| 32 | 79 | | Ascending thoracic | - | OSR | 2018 | HCA | M | GP | *S. agalactiae* | SSTI | 5.37 | 1 | 1 | 1 | Yes^(-)^ | No | No | Yes | No |
| 33 | 67 | | Abdominal | - | EVAR | 2019 | HCA | M | GN | *E. coli* | Abdominal | 7.08 | 2 | 4 | 1 | No | No | No | No**^d^** | No |
| 34 | 74 | | Abdominal | - | OSR | 2019 | HCA | M | GN | *E. coli* | Urinary tract | 0.05 | 2 | 4 | 1 | No | No | No | Yes | No |
| 35 | 71 | | Abdominal | - | EVAR | 2012 | HCA | M | GP | *S. pneumoniae* | Pulmonary | 0.72 | 1 | 2 | 1 | No | No | No | Yes | No |
| 36 | 57 | | Abdominal | - | OSR | 2017 | NO | P | GN | *B. thetaiotaomicron/faeces,*  *E. lenta, B. fragilis* | Abdominal | 0.01 | 4 | 4 | 2 | Yes^(-)^ | No | No | Yes | No |
| 37 | 73 | | Abdominal | AV | EVAR | 2020 | HCA | M | GP | *F. magna* | Unknown | 7.89 | 1 | 1 | 1 | No | No | No | Yes | No |
| 38 | 82 | | Abdominal | CIED | EVAR | 2019 | HCA | M | GP | *MSSA* | Unknown | 0.10 | 15 | 22 | 7**^b^** | No | No | Yes^(-)^ | Yes | No |
| 39 | 75 | | Descending thoracic | - | EVAR | 2015 | NO | - | GN | *K. pneumoniae* | Urinary tract | 0.04 | 2 | 5 | 1 | No | No | No | Yes | No |
| 40 | 74 | | Ascending thoracic | AV | OSR | 2015 | NO | M | GN | *P. aeruginosa* | Pulmonary | 0.03 | 2 | 3 | 1 | No | No | No | No**^d^** | No |
| 41 | 84 | | Abdominal | - | EVAR | 2015 | HCA | P | Both | *P. aeruginosa, A. schaalii* | Urinary tract | 4.38 | 2 | 4 | 1 | No | No | No | Yes | No |
| 42 | 62 | | Ascending thoracic | AV**^a^**, MV**^a^** | OSR | 2018 | CA | M | GP | *S. mitis group* | Unknown | 2.20 | 8 | 14 | 3**^b^** | Yes^(-)^ | No | No | Yes | No |

**Abbreviations: AV**: Aortic valve; **CIED**: Cardiovascular implantable electronic device**; CA**: community-onset; **CT-A**: computed tomography with angiogram; **EVAR**: Endovascular repair; **GN**: Gram-negative; **GP**: Gram-positive; **HCA**: Healthcare-associated; **I^111^**: 111 indium white blood cell scan; **M**: Monomicrobial; **MSSA**: Methicillin-susceptible *S. aureus*; **MV**: Mitral valve; **NO**: Nosocomial; **OSR**: Open surgical repair; **P**: Polymicrobial; **PET-CT**: Positron emission tomography with computed tomography.

**^a^** Device considered infected.

**^b^** Persistent BSI.

**^C^** Lost to follow-up less than 14 days from BSI.

**^d^** Died less than 14 days from BSI.

**^e^** Suspected VGI.

**^f^** Confirmed VGI.

^(+)^ Findings are suggestive of possible VGI (refer to MAGIC radiographic criteria).

^(-)^ No findings suggestive of VGI (refer to MAGIC radiographic criteria).

| Supplementary Table 3. MAGIC criteria fulfilled by VGI Cases. | | | | | | | | | | |
| --- | --- | --- | --- | --- | --- | --- | --- | --- | --- | --- |
|  | **Patient ID** | | | | | | | | | |
|  | **1** | **2** | **3** | **4** | **5** | **6** | **7** | **8** | **9** | **10** |
| Magic major criteria |  | | | | | | | | | |
| Pus (definite by microscopy) around graft or aneurysm sac at surgery |  |  | + |  | + |  |  |  |  |  |
| Open wound with exposed graft or communicating sinus |  |  |  |  |  |  |  |  |  |  |
| Fistula development (e.g., aorto-enteric or aortobronchial) |  |  |  |  |  |  |  |  |  |  |
| Graft insertion in an infected site (e.g., fistula, mycotic aneurysm, or infected pseudo-aneurysm) |  |  |  |  |  |  |  |  |  |  |
| Perigraft fluid on CT scan ≥ 3 months after insertion |  |  |  | + |  | + |  |  | + | + |
| Perigraft gas on CT scan ≥ 7 weeks after insertion |  |  |  |  |  |  |  |  |  |  |
| Increase in peri-graft gas volume demonstrated on serial imaging |  |  |  |  |  |  |  |  |  |  |
| Microorganism recovered from an explanted graft |  |  | + | + |  |  |  |  |  |  |
| Microorganisms recovered from an intra-operative specimen |  |  | + | + |  |  |  |  |  |  |
| Microorganisms recovered from a percutaneous aspirate of peri-graft fluid |  |  |  | + |  |  |  |  | + |  |
| Magic minor criteria |  |  |  |  |  |  |  |  |  |  |
| Localized clinical features of VGI (e.g., erythema, warmth, swelling, purulent discharge, and pain) |  |  |  |  |  |  |  |  |  |  |
| Fever ≥ 38◦C with VGI as the most likely cause |  | + |  | + | + | + | + | + | + |  |
| Other suspicious radiographic findings | + | + | + | + | + | + | + | + | + | + |
| Blood culture(s) positive and no apparent source except for VGI | + | + |  | + |  | + | + | + | - | + |
| Abnormally elevated inflammatory markers with VGI as the most likely cause (e.g., ESR, CRP, and white cell count) | + | + | + | + | + | + | + | + | + | + |
| Interpretation | S | S | C | C | C | C | S | S | C | C |

The patient ID in this table corresponds with the patient ID found in **Supplementary Table 2**.

**Abbreviations: C**: Confirmed VGI; **S**: Suspected VGI.

| Supplementary Table 4. Matching Blood Culture Isolates with Graft and Peri-graft Cultures. | | | | | |
| --- | --- | --- | --- | --- | --- |
| Patient ID | Blood culture result | Surgical explantation | Image-guided aspiration | Graft culture result | Aspirate culture |
| 3 | *Bacteroides uniformis* | Yes | - | *Bacteroides uniformis* | - |
| 4 | *Pseudomonas aeruginosa* | Yes | - | *Pseudomonas aeruginosa* | - |
| 5 | *Streptococcus mitis* group | Yes | - | Negative | - |
| 9 | MSSA | - | Yes | - | MSSA, *Mycoplasma hominis* |

The patient ID in this table corresponds with the patient ID found in **Supplementary Table 2**.

**Abbreviations: MSSA:** Methicillin-susceptible *Stapylococcus* *aureus*.

**Follow-up:**

The observational units in the study are arterial aneurysm repairs, with some patients requiring multiple procedures during the study period. We considered each graft repair at a unique site of aneurysm per patient to be at risk for developing subsequent BSI. For all patients, follow-up for BSI initially started at the time of their first aneurysm repair. When patients underwent a subsequent repair at the same site, their initial graft follow-up was stopped, and their subsequent graft follow-up restarted at the time of their second repair. On the other hand, when patients underwent a subsequent aneurysm repair at a different site, the follow-up for their initial procedure continued to accrue and could run concurrently with the follow-up from the subsequent procedure. For mortality, the follow-up of each index procedure was truncated at the subsequent aneurysm repair regardless of site, to ensure that those patients contributed distinct intervals of follow-up across multiple records. At the patient level, follow-up was terminated at their last encounter or at the time of the outcome.
